# Subjects with Discordant Airways Obstruction: Lost between Spirometric Definitions of COPD

**DOI:** 10.1155/2011/780215

**Published:** 2011-02-06

**Authors:** Bernd Lamprecht, Lea Schirnhofer, Bernhard Kaiser, Sonia A. Buist, David M. Mannino, Michael Studnicka

**Affiliations:** ^1^Department of Pulmonary Medicine, Paracelsus Private Medical University of Salzburg, 5020 Salzburg, Austria; ^2^Department of Pulmonary and Critical Care Medicine, Oregon Health and Science University, Portland, OR 97239, USA; ^3^Department of Preventive Medicine and Environmental Health, University of Kentucky, Lexington, KY 40536, USA

## Abstract

*Background*. Since the FEV1/FVC ratio declines with age, using the fixed ratio of 0.70 leads to overdiagnosis of COPD in older populations and underdiagnosis among young adults. *Objective*. To evaluate whether *discordant obstructive cases* (FEV1/FVC < 0.70 but ≥LLN) are a healthy population or have clinical features that would place them at increased risk. *Methods*. We used post-bronchodilator spirometry data from the population-based Austrian Burden of Obstructive Lung Disease (BOLD) study. Those with post-bronchodilator FEV1/FVC ratio <LLN and <0.70 were defined as *concordant obstructive cases*. Participants with post-bronchodilator FEV1/FVC ratio ≥LLN but <0.70 were defined as *discordant obstructive cases*. *Results*. *Discordant obstructive cases* were more likely to be older, male and never-smokers. Additionally they had less respiratory symptoms and less severe impairment of FEV1. However, *discordant obstructive cases* reported significantly more often a diagnosis of heart disease than subjects with normal lung function (27.2% vs 7.3%, *P* = .015). *Conclusion*. The clinical profile of discordant obstructive cases includes potentially important comorbid disease.

## 1. Introduction

Lung function measurements are recommended as essential for diagnosing COPD and helpful for grading its severity [1].

According to current GOLD guidelines [2], chronic obstructive pulmonary disease is defined as airflow limitation with a postbronchodilator FEV1/FVC ratio below 70%. 

On the one hand, these criteria have simplified the diagnosis of COPD and have helped to improve the awareness of this disease. But on the other hand, the use of a fixed threshold to define airways obstruction is associated with some extent of misclassification. Since the FEV1/FVC ratio declines with age, there is a risk of overdiagnosis of COPD among healthy elderly people [3]. Because of this misclassification problem, the American Thoracic Society/European Respiratory Society guidelines [4] continue to officially recommend an interpretative algorithm that uses the 5th percentile lower limit of normal (LLN), which is age-specific and declines with age, to define pulmonary function abnormalities.

For this study, we analyzed postbronchodilator spirometry data of an Austrian population-based survey in adults aged 40 years and over. We compared the fixed ratio with the lower limit of normal as a diagnostic criterion for nonreversible airways obstruction. We tested the hypothesis that *discordant obstructive cases* (FEV1/FVC < 0.70 and ≥LLN) are a healthy population (i.e., they are elderly, asymptomatic subjects without the presence of known risk factors for chronic airways obstruction) or whether these in-between cases might represent subjects at risk who should be followed carefully.

## 2. Methods

### 2.1. Study Population

The study population consisted of participants of the Austrian Burden of Obstructive Lung Disease (BOLD) study [5]. In this study, a gender-stratified random sample of the inhabitants of Salzburg County, aged 40 years and over, was surveyed. Of the 2,200 individuals (1,100 men and 1,100 women) whom we attempted to contact, 1,258 (60%) completed the full protocol and had spirometry findings that met ATS quality control criteria (see below). These individuals constitute the primary sample for this analysis. The study was approved by the local Ethics Committee of Salzburg County, and all participants gave written informed consent.

### 2.2. Study Measures

Spirometry was done according to American Thoracic Society (ATS) criteria [6] by trained and certified technicians using the NDD Easy One spirometer with participants in a seated position. Separate measurements were made before and at least 15 minutes after two puffs of salbutamol (200 *μ*g) metered dose inhaler, administered using a Volumatic spacer. Spirometry data were sent electronically to the Pulmonary Function Quality Control Centre in Salt Lake City, Utah, USA, where each spirogram was reviewed and graded using ATS guidelines [6]. 

Only spirograms that met ATS acceptability and reproducibility criteria were included; at least three trials, two acceptable (free from artefact, sudden stops, and back extrapolated volumes greater than 5.0% of FVC) and reproducible (difference between largest and second largest values is less than 200 mL) tests for both the forced expiratory volume in one-second (FEV_1_) and the forced vital capacity (FVC). Study technicians were continuously monitored. When a technician's quality score dropped below a preset level, he/she had to stop testing and be retrained and recertified.

### 2.3. Questionnaire Data

The BOLD questionnaires included information on respiratory symptoms, risk factors for COPD, comorbidities, and respiratory diagnoses, and were administered by a trained and certified staff.

### 2.4. Definitions

Nonreversible “*airways obstruction*” was defined either as a postbronchodilator FEV1/FVC ratio below the lower limit of normal (LNN) or an FEV1/FVC ratio below 0.70 (according to GOLD criteria). 

The NHANES III reference equations [7] were used to calculate predicted values and LLNs for FEV1, FVC, and FEV1/FVC. The Lower limit of normal (LLN) is based on the lower fifth percentile of the index.

In the presence of an abnormal FEV1/FVC ratio, “*severity of airway obstruction*” was graded as follows, possible normal variant (FEV1 > 100% predicted), mild (FEV1 70–100% predicted), moderate (FEV1 50–70% predicted), and severe (FEV1 < 50% predicted).

Participants whose postbronchodilator FEV1/FVC ratio was below both the LLN and the fixed ratio of 0.70 were defined as “*concordant obstructive cases*”.

Participants who had a postbronchodilator FEV1/FVC ratio above the LLN but below the fixed ratio of 0.70 were defined as “*discordant obstructive cases*”.

“*Restrictive lung function*” was defined as postbronchodilator FVC < 80% predicted and a FEV1/FVC > 70%. 

“Doctor-diagnosed COPD” was defined as self-reported physician's diagnosis of chronic bronchitis, emphysema, or COPD.

“*Ever smoking*” (current or former smoking) was defined as smoking more than 20 packs of cigarettes in a lifetime or more than 1 cigarette/day for a year. “*Never smoking*” was defined as smoking less than 20 packs of cigarettes in a lifetime.

Additional measures evaluated included self-report of respiratory symptoms for “*cough*” (*Do you usually cough when you don't have a cold?*), “*phlegm*” (*Do you usually bring up phlegm from your chest, or do you usually have phlegm in your chest that is difficult to bring up when you don't have a cold?*), and “*dyspnea*” (*Are you troubled by shortness of breath when hurrying on the level or walking up a slight hill?);* and “self-reported physician diagnosis” of asthma, tuberculosis, heart disease, hypertension, diabetes, and stroke.

### 2.5. Statistical Analysis

Sensitivity and specificity of FEV1/FVC < LLN for diagnosing airway obstruction defined by FEV1/FVC < 0.70 were calculated using 2 × 2 tables. Statistical significance of differences was evaluated using the chi-square test and the nonparametric Wilcoxon test. All statistical analyses were done with SAS 8.2 (SAS Institute, Inc., Cary, NC).

## 3. Results

Of the 1349 participants with postbronchodilator spirometry, 1258 (93%) met the quality control criteria and were included in this analysis. Population characteristics and postbronchodilator lung function data of the study population (*n* = 1258) are shown in Table 1.

Applying the current GOLD criteria (FEV1/FVC < 0.70) to postbronchodilator spirometry, the prevalence of COPD GOLD stage I or higher in our population-based sample of adults aged 40 years and over was 24.2%. In contrast to this, utilization of the lower limit of normal as a threshold for the FEV1/FVC ratio (FEV1/FVC < LLN) demonstrated a prevalence of 15.3%. Prevalence of doctor-diagnosed COPD (reported physicians diagnosis of COPD, emphysema, or chronic bronchitis) was 5.6%.

**Figure 1:**
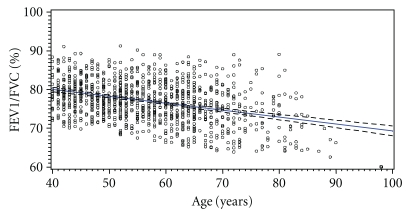
Study subjects with FEV1/FVC > LLN (*n* = 1059): relationship between FEV1/FVC% and age. Lines represent regression of FEV1/FVC ratio by age (solid line) with 95% confidence limits for mean predicted values (dotted lines). The horizontal line indicates the 70% level of the FEV1/FVC ratio.

**Figure 2:**
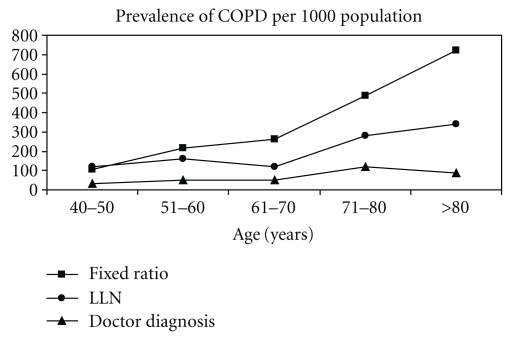
Prevalence of nonreversible airways obstruction with age, using different definitions (per 1000 population). “Fixed ratio”: postbronchodilator FEV1/FVC < 0.70, “LLN”: postbronchodilator FEV1/FVC < LLN, “doctor diagnosis”: prior physicians diagnosis of COPD.

Figure 1 shows the declining FEV1/FVC ratio by age in the study group without airways obstruction (*n* = 1059, FEV1/FVC > LLN). The prevalence of COPD by diagnostic definition and age group is illustrated in Figure 2. Sensitivity and specificity of the widely used fixed ratio (0.70) compared to the lower limit of normal are shown in Table 2.

Concordant obstructive cases (FEV1/FVC < 0.70 & <LLN) showed a trend towards more severe airways obstruction than discordant obstructive cases (FEV1/FVC < 0.70 & ≥LLN), see Table 3. 89.3% of discordant obstructive cases had a FEV1 greater than 70% of its predicted value, and none of the discordant obstructive cases had a FEV1 less than 50% of its predicted value.

Overall, 6.4% (*n* = 81) of the study population were discordant obstructive cases (FEV1/FVC < 0.70 & ≥LLN) with mild to moderate obstructive impairment (FEV1 100–50% predicted). Concordant obstructive and discordant obstructive cases with mild to moderate impairment of FEV1 differed significantly in terms of age, gender, and reported cough and phlegm (see Table 4). However, when adjustments for age were made, they were very similar in terms of comorbid disease, smoking status, and reported dyspnea (see Table 4).

The clinical profile of subjects with discordant obstructive findings was similar to the profile of subjects with restrictive lung function (see Table 4). In addition to this, subjects with discordant obstructive findings (FEV1/FVC < 0.70 & ≥LLN) reported significantly more often a diagnosis of heart disease than subjects with “normal” lung function (FEV1/FVC > 0.70 & FVC ≥ 80% pred.), see Table 4.

## 4. Discussion

This population-based study contributes to the characterization of discordant obstructive cases (=subjects with “normal” findings using the LLN but abnormal findings using the fixed cutoff of 70% as a threshold for the FEV1/FVC ratio). The data suggest that subjects who are in between the two definitions of airways obstruction may not have clinically significant airways obstruction. However, their clinical profile is characterized by relevant comorbid disease and is clearly different from the profile of age-matched subjects with “normal” lung function.

### 4.1. The Discrepancy between the Different Definitions

The discrepancy in the prevalence of airways obstruction depending on the criteria used to define disease is consistent with other studies.

A similar difference in the prevalence of COPD depending on the definition used was reported for adults aged 40 years and over in New Zealand, showing a GOLD-defined prevalence of COPD of 14.2% and a LLN-defined prevalence of 9.0% [8]. A recent study based on spirometry tests from a regional primary care diagnostic centre in the Netherlands has shown that the discrepancy between the two definitions for airflow obstruction increases along with age, and that utilization of the fixed FEV1/FVC cutoff may cause substantial COPD overdiagnosis in primary care [9]. In an elderly Chinese population (subjects aged 60 years and above), the prevalence of airways obstruction using the fixed ratio of 0.70 or using the LLN for the postbronchodilator FEV1/FVC ratio was 25.9% and 12.4%, respectively [10].

A population-based study in Norway using pre-bronchodilator spirometry data has shown that a FEV1/FVC ratio below 0.70 was 50% more frequent in women aged 70 years and above compared to women aged 60–69 years. The corresponding increase in men was 80%. On the basis of their results, the authors suggested the use of a threshold of 65% (FEV1/FVC < 0.65) for subjects over the age of 70 years [11].

However, the discrepancy depending on the definition used shows that either the GOLD criteria lead to a relevant percentage of false positives or utilization of the lower limit of normal to a relevant percentage of false negatives.

In general, any diagnostic test should have both high sensitivity and high specificity. Since the FEV1/FVC ratio declines with age, the fixed ratio of 0.70 which is recommended by the GOLD Initiative, may lead to considerable overdiagnosis among healthy elderly people and underdiagnosis among young adults with early disease [3, 12–15]. The problem of misclassification might be pronounced in the primary care setting, where early stages of disease are seen. Therefore, utilization of the lower limit of normal instead of a fixed ratio has been especially recommended for COPD screening in primary care [16].

### 4.2. Pros and Cons of the Different Definitions

While utilization of the fixed ratio (0.70) is simple, convenient, and independent from the existence of adequate reference equations and specially adapted spirometers, the use of the age- and sex-specific LLN as a threshold for the FEV1/FVC ratio may lessen the risk of overdiagnosis of airways obstruction among the elderly and underdiagnosis among young adults. 

Utilization of GOLD guidelines and unreflective interpretation of test results may have negative consequences by misclassifying healthy elderly subjects as COPD and thus possibly causing unnecessary treatment and healthcare costs. With this and other critical issues in mind, a growing number of experts recommend to replace the 0.70 threshold with the lower limit of normal threshold [17].

### 4.3. Discordant Obstructive Cases (In-Between Cases)

When one definition of airways obstruction (FEV1/FVC < LLN) is thought to replace another definition (FEV1/FVC < 0.70) the main point of interest is of course the number and characteristics of those subjects who have discordant findings (“normal” using the LLN but “abnormal” using the fixed ratio).

The comparison of concordant obstructive (FEV1/FVC < 0.70 & <LLN) cases and discordant obstructive (FEV1/FVC < 0.70 & ≥LLN) cases revealed that subjects in the latter group were significantly older and significantly more likely to be male. In addition to this, discordant obstructive cases reported less active smoking and less respiratory symptoms. Our data suggest that utilization of the LNN as a threshold for the FEV1/FVC ratio helps to identify clinically significant disease.

However, the clinical profile of cases in between the two definitions (discordant obstructive cases FEV1/FVC < 0.70 & ≥LLN) was characterized by high amounts of comorbid disease. When adjustments for age were made, subjects with discordant obstructive findings reported significantly more often a diagnosis of heart disease than subjects with normal lung function. The clinical profile of subjects with discordant obstructive findings was very similar to the profile of subjects with restrictive lung function. Therefore, this data suggest that a proportion of individuals with discordant obstructive findings may have clinically important comorbidities. Results of a cohort study of the Cardiovascular Health Study have shown that subjects classified as “normal” using the LLN but abnormal using the GOLD criteria (0.70) were more likely to die and to have a COPD-related hospitalization during followup. The authors suggested that a fixed ratio of less than 0.70 identifies at-risk patients, even among older adults [18]. In an editorial on the definition of COPD the author suggested that a finding that predicts a bad event or premature death may probably represent “disease” [19].

### 4.4. Definition of Airways Obstruction and Underdiagnosis

Besides the valuable discussion on what might be the most precise definition of airways obstruction, one has to take into consideration that currently the majority of disease is still undiagnosed and untreated. The prevalence of physician-diagnosed chronic airways obstruction in our study population was 5.6%. This is far below both the prevalence defined by postbronchodilator FEV1/FVC **<** 0.70 (24.2%) and FEV1/FVC **<** LLN (15.3%). A recent study in a primary care setting in Poland has shown that only 18.6% of subjects with COPD had previously been diagnosed [20]. Overall, about 8%–13% of people in general population surveys have airways obstruction without having a diagnosis of obstructive lung disease [21].

### 4.5. Limitations

Limitations of our study include the use of reference equations from the United States that may not ideally apply to our population. However, using either the NHANES III predictions or predictions based on the study sample gave the same high COPD prevalence [5]. Another potential limitation of our study is that 200 mcg of salbutamol was administered instead of 400 mcg, as recommended by the GOLD guidelines. The prevalence of post-BD airway obstruction would have been slightly lower with a higher dose of BD. However, many pulmonary function laboratories have been using 200 mcg and we worried about the risk of arrhythmias in our field settings. The timing of the postbronchodilator spirometry only 15 minutes after the administration of salbutamol, while standard practice, may nonetheless underestimate the true bronchodilator response which may not be evident for 30–60 minutes.

## 5. Conclusion

The lower limit of normal (LLN) seems to be the more reasonable threshold to define clinically significant airways obstruction. The risk of substantial overdiagnosis of COPD and subsequent unnecessary treatment and healthcare costs (due to utilization of a simple fixed threshold) is pitted against the reasonable desire for simplicity.

The results of this study indicate that subjects who are in between the two definitions of airways obstruction (discordant obstructive cases, FEV1/FVC < 0.70 & ≥LLN) do not have clinically significant airways obstruction. However, their clinical profile is characterized by relevant comorbid disease and, therefore, they might be at risk and should be followed carefully.

## Figures and Tables

**Table 1:** Population characteristics and postbronchodilator lung function data (*n* = 1258).

	Male	Female
Subjects, *n* (%)	685 (54.5%)	573 (45.5%)
Age distribution yrs, *n* (%)		
40–50	206 (30.1%)	183 (31.9%)
51–60	202 (29.5%)	175 (30.5%)
61–70	169 (24.7%)	136 (23.7%)
71–80	83 (12.1%)	57 (10.0%)
81+	25 (3.6%)	22 (3.9%)

Body mass index [kg/(m^2^)], mean (SD)	26.7 (0.14)	26.1 (0.20)
FVC (L), mean (%pred.)	4.5 (97.5%)	3.4 (100.6%)
FEV1 (L), mean (%pred.)	3.4 (95.0%)	2.5 (95.9%)
FEV1/FVC%, mean (%pred.)	74.1 (97.6%)	74.5 (95.5%)

**Table 2:** Airways obstruction defined by postbronchodilator FEV1/FVC < 0.70 and FEV1/FVC < LLN.

	FEV1/FVC < 0.70 (**n** = 304)	FEV1/FVC ≥ 0.70 (**n** = 954)
FEV1/FVC < LLN (**n** = 199)	192 (63.2%)	7 (0.7%)
FEV1/FVC ≥ LLN (**n** = 1059)	112 (36.8%)	947 (99.3%)

**Table 3:** Severity of airways obstruction in concordant and discordant obstructive cases.

Severity of airways obstruction	Concordant obstructive cases^§^ (*n* = 192)	Discordant obstructive cases^#^(*n* = 112)	*P*-value
Possible normal variant (FEV1 >100% predicted)	22 (11.4%)	31 (27.7%)	<.001
Mild obstruction (FEV1 70%–100% predicted)	115 (59.9%)	69 (61.6%)	<.001
Moderate obstruction (FEV1 50%–70% predicted)	41 (21.4%)	12 (10.7%)	.003
Severe obstruction (FEV1 < 50% predicted)	14 (7.3%)	0 (0.0%)	/

^§^Concordant obstructive cases: FEV1/FVC < 0.70 & <LLN.

^#^Discordant obstructive cases: FEV1/FVC < 0.70 & ≥LLN.

**Table 4:** Characteristics of subjects with discordant obstructive findings and comparison to subjects with normal lung function, subjects with restrictive lung function and subjects with concordant obstructive findings. Presented obstructive cases are limited to those with mild to moderate impairment of FEV1 (FEV1 50%–100% pred.).

	Discordant obstructive cases^#^ (*n* = 81)	“Normal” lung function^$^ (*n* = 414)	Restrictive lung function^Ψ^ (*n* = 82)	Concordant obstructive cases^§^ (*n* = 156)	*P*-value*	*P*-value**	*P*-value***
*Characteristics*							
Age, mean (SE)	67 (0.99)	54.8 (0.50)	59.4 (1.16)	60.5 (0.95)	<.001	<.001	<.001
Sex, male	66 (81.5%)	230 (55.6%)	48 (58.5%)	76 (48.7%)	<.001	.002	<.001
Never smoker	33 (40.7%)	203 (49.0%)	35 (42.7%)	45 (28.9%)	.172	.802	.065

*Respiratory Symptoms* ^*ϕ*^							
Cough	14 (17.3%)	60 (14.5%)	19 (23.2%)	51 (32.7%)	.796	.291	.009
Phlegm	20 (24.7%)	88 (21.3%)	23 (28.1%)	65 (41.7%)	.893	.442	.004
Dyspnea	24 (29.6%)	62 (15.0%)	29 (35.4%)	41 (26.3%)	.085	.084	.987

*Comorbidities* ^*ϕ*^							
Asthma	7 (8.6%)	29 (7.0%)	5 (6.1%)	21 (13.5%)	.116	.172	.585
Heart disease	22 (27.2%)	30 (7.3%)	19 (23.2%)	23 (14.7%)	.015	.179	.450
Hypertension	33 (40.7%)	135 (32.6%)	29 (35.4%)	55 (35.3%)	.223	.970	.689
Diabetes	10 (12.4%)	32 (7.7%)	8 (9.8%)	10 (6.4%)	.661	.538	.343
Stroke	6 (7.4%)	6 (1.5%)	4 (4.9%)	3 (1.9%)	.228	.643	.156
Tuberculosis	2 (2.5%)	10 (2.4%)	2 (2.4%)	8 (5.1%)	.479	.929	.195

^#^Discordant obstructive cases: FEV1/FVC < 0.70 & ≥LLN.

^§^Concordant obstructive cases: FEV1/FVC < 0.70 & <LLN.

^$^Normal lung function: FEV1/FVC > 0.70 & FVC ≥ 80% pred.

^Ψ^Restrictive lung function: FEV1FVC > 0.70 & FVC < 80% pred.

^*ϕ*^Results adjusted for age.

*P-value for difference between “discordant obstructive cases” and “normal lung function”.

**P-value for difference between “discordant obstructive cases” and “restrictive lung function”.

***P-value for differences between “discordant obstructive cases” and “concordant obstructive cases”.
